# Supplementary material for: Development and Evaluation of EDTA-Treated Rabbits for Bioavailability Study of Chelating Drugs Using Levofloxacin, Ciprofloxacin, Hemiacetal Ester Prodrugs, and Tetracycline
Source: Pharmaceutics. 2023 May 24;15(6):1589. doi: 10.3390/pharmaceutics15061589 (PMC10301896; doi:10.3390/pharmaceutics15061589)
Supplement: Supplementary file 1 [file pharmaceutics-15-01589-s001.zip › pharmaceutics-2393226-supplementary.pdf]

Additional files

(A)

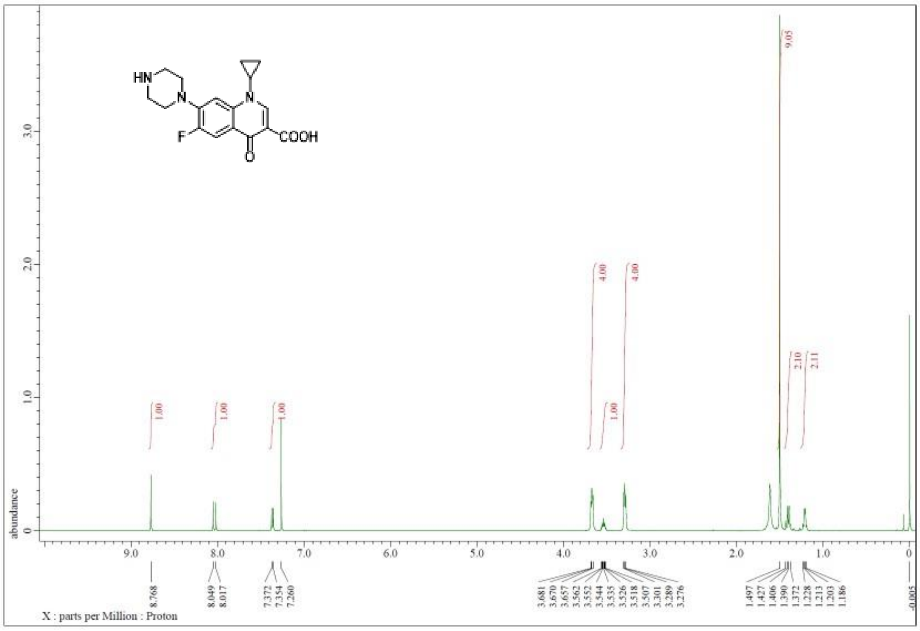

(B)

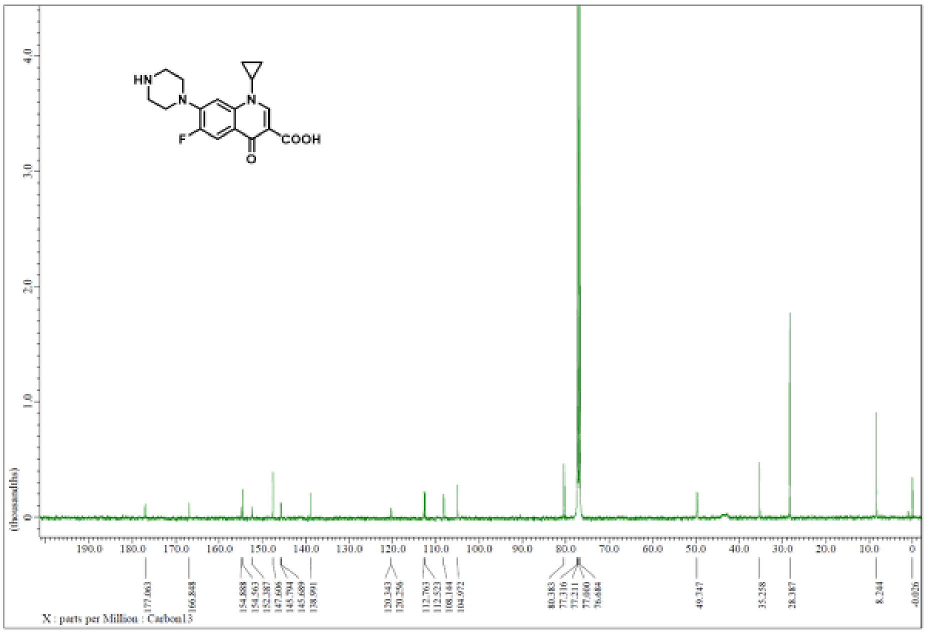

Figure S1. Spectrum of ^{1}H-NMR (A), and ^{13}C-NMR (B) of CFX

(A)

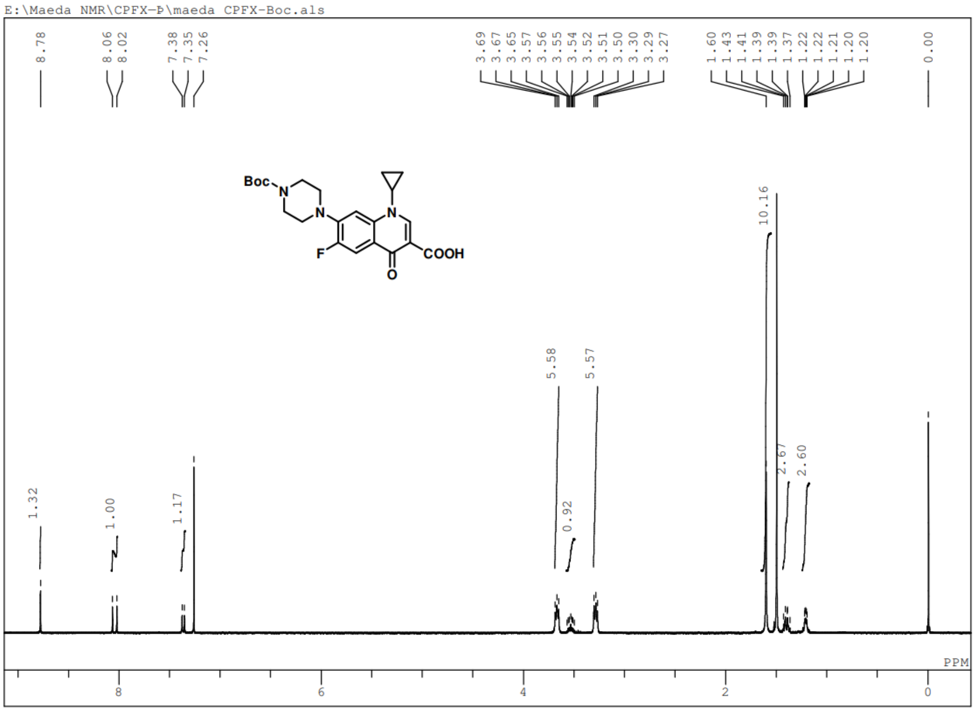

(B)

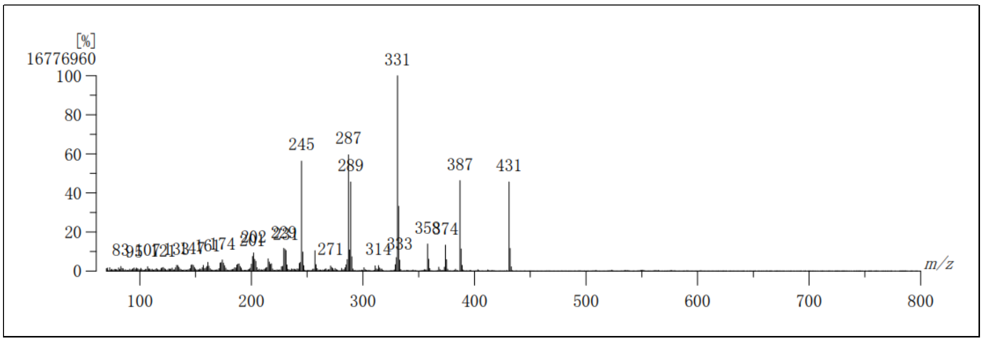

Figure S2. Spectrum of ^{1}H-NMR (A), and Mass (B) of Boc-CFX

(A)

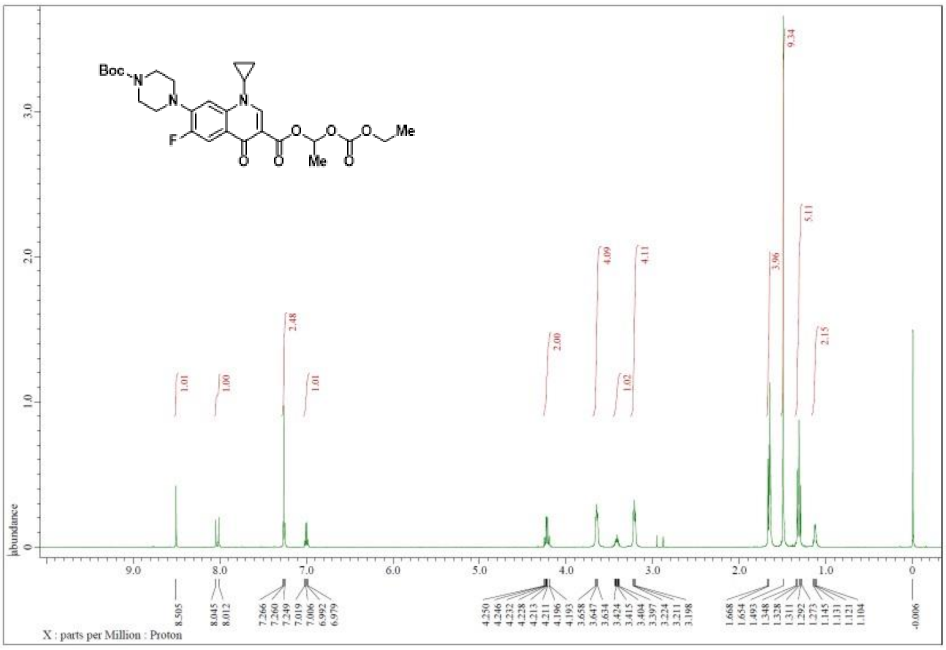

Boc
N
N
F
O
O
O
O
O
O
Me
Me
abundance
X : parts per Million : Proton

(B)

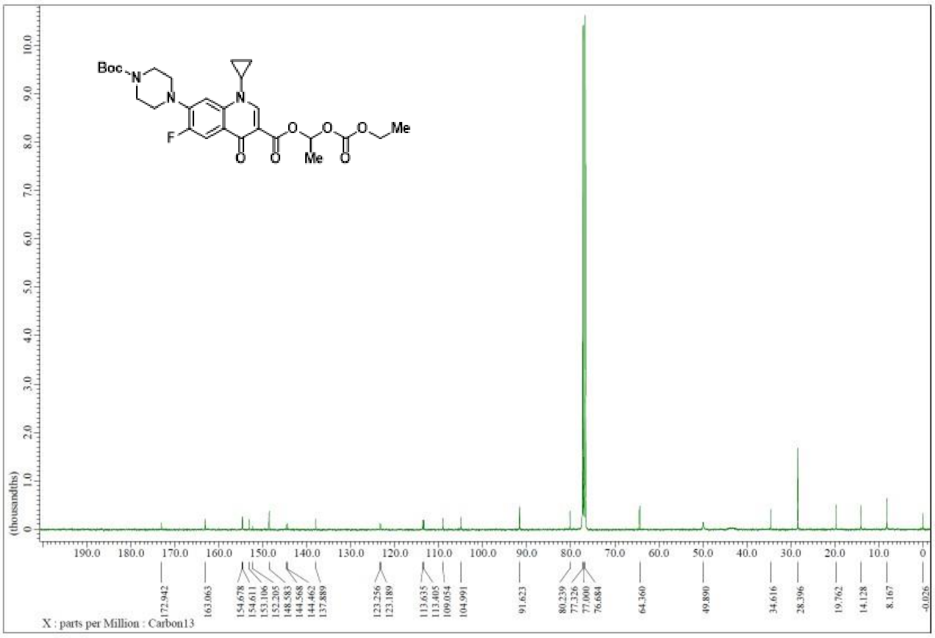

Boc
N
N
F
O
O
O
O
O
O
Me
Me
(thousandths)
X : parts per Million : Carbon13

(C)

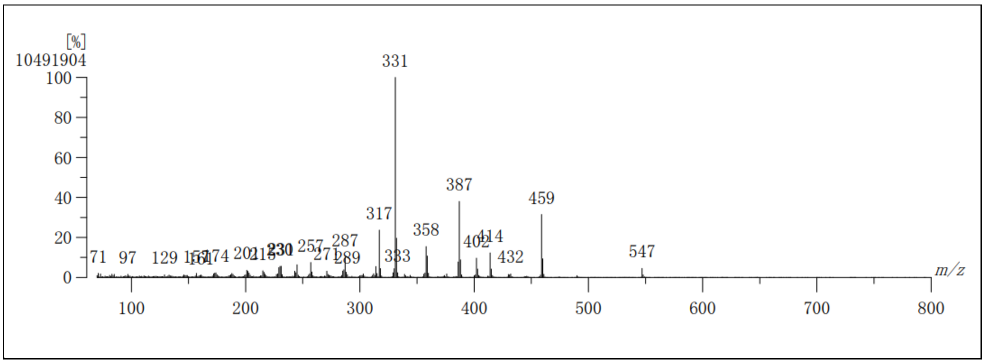

Figure S3. Spectrum of ^{1}H-NMR (A), ^{13}C-NMR (B) and Mass (C) of Boc-CFX-EHE

(A)

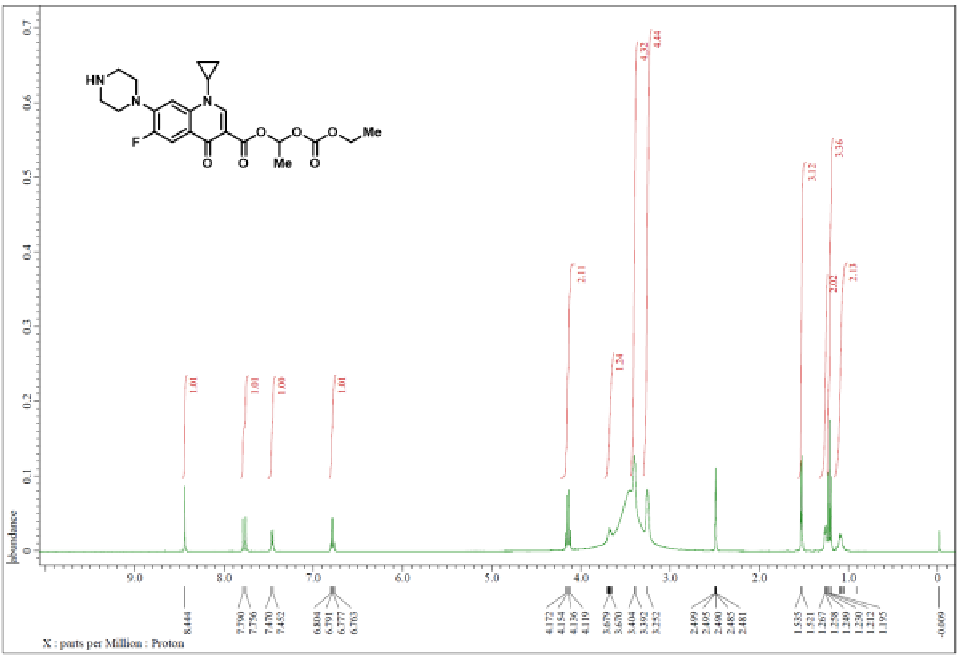

(B)

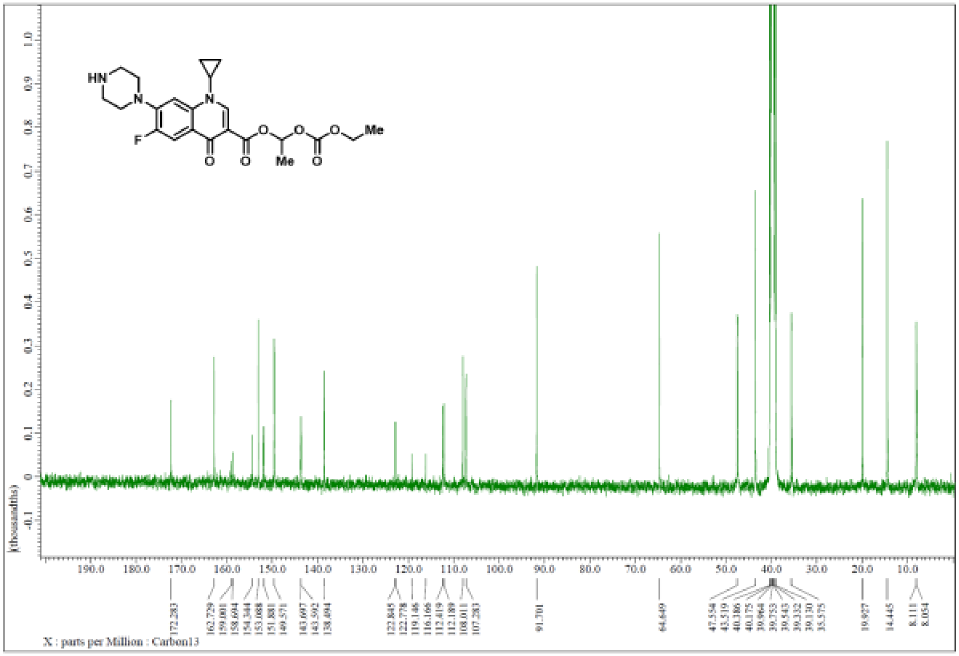

(C)

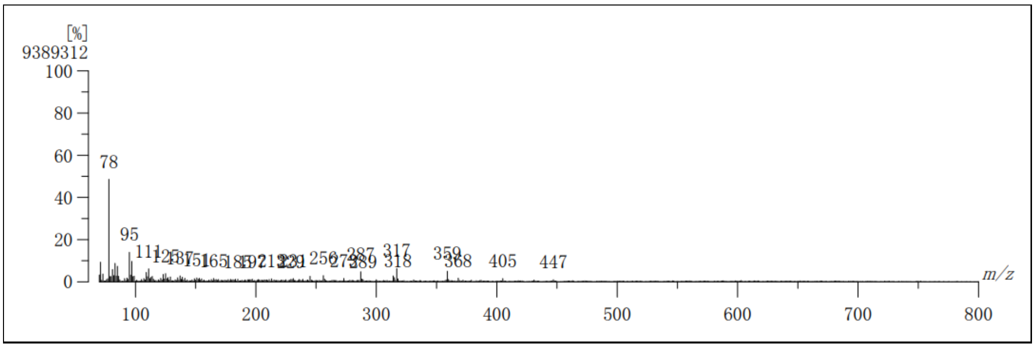

Figure S4. Spectrum of ^{1}H-NMR (A), ^{13}C-NMR (B) and Mass (C) of CFX-EHE
